# A novel room-temperature CQD fluorescent nanosensor for the first derivatization-free spectrofluorimetric determination of dalfampridine: application to biological fluids and content uniformity testing

**DOI:** 10.1039/d5ra09768a

**Published:** 2026-03-16

**Authors:** Rehab H. Elattar, Manal A. Alossaimi, Ahmed Emad F. Abbas, Anmar Anwar Khan, Bodour S. Rajab, Mazen M. Ghaith, Ahmed H Qasem, Galal Magdy

**Affiliations:** a Pharmaceutical Analytical Chemistry Department, Faculty of Pharmacy, Suez Canal University Ismailia Egypt rehabhamdy216@gmail.com; b Pharmaceutical Chemistry Department, College of Pharmacy, Prince Sattam bin Abdulaziz University Al-Kharj 11942 Saudi Arabia; c Analytical Chemistry Department, Faculty of Pharmacy, October 6 University 6 October City Giza 12585 Egypt; d Department of Clinical Laboratory Sciences, Faculty of Applied Medical Sciences, Umm Al-Qura University Makkah 21955 Saudi Arabia; e Pharmaceutical Analytical Chemistry Department, Faculty of Pharmacy, Kafrelsheikh University Kafrelsheikh 33511 Egypt galal_magdy@pharm.kfs.edu.eg; f Department of Pharmaceutical Analytical Chemistry, Faculty of Pharmacy, Mansoura National University Gamasa, 7731168 Egypt

## Abstract

This study presents a novel approach for producing luminescent carbon quantum dots (CQDs) at room temperature (RT) utilizing hydroquinone and monosodium glutamate (MSG) as precursors through a simple reaction with no energy consumption. The produced RT-CQDs have a tiny diameter of 3.6 nm and a high quantum yield of 38.05%. Many spectroscopic and microscopic techniques, including HRTEM, zeta potential, EDX, FT-IR, UV/Visible, and fluorescence spectroscopy were utilized to characterize the produced RT-CQDs. After excitation at 290 nm, the RT-CQDs showed a high emission at 327 nm. The produced RT-CQDs' fluorescence signal was quantitatively decreased by dalfampridine (DFP), enabling their use as a sensitive fluorescent nanosensor to measure DFP in different matrices without the need for any pre-derivatization steps or a large volume of organic solvents, for the first time. The developed method showed a correlation coefficient of 0.9998, wide linearity range of 0.02–14.0 µg mL^−1^, and a detection limit of 0.005 µg mL^−1^, indicating the method's exceptional sensitivity. The proposed method was effectively used to determine DFP in tablets and human plasma samples, with low% RSD and high% recoveries. Moreover, the DFP tablets' content uniformity testing was carried out in compliance with USP criteria. Furthermore, the newly published greenness, blueness, and violetness assessment tools proved the low environmental effect, excellent practicality, good analytical performance, and novelty of the proposed DFP sensing approach. The developed method was fully validated according to ICH guidelines. The production of CQDs with superior qualities at room temperature under mild conditions opens the door for further developments in CQD room temperature synthesis and provides an effective substitute for conventional synthesis methods.

## Abstract 2

Hydroquinone and monosodium glutamate were first used to synthesize room-temperature carbon quantum dots (RT-CQDs). The fluorescent RT-CQDs acted as a switch-off nanoprobe for sensitive dalfampridine analysis in pharmaceuticals and human plasma.

## Graphical abstract

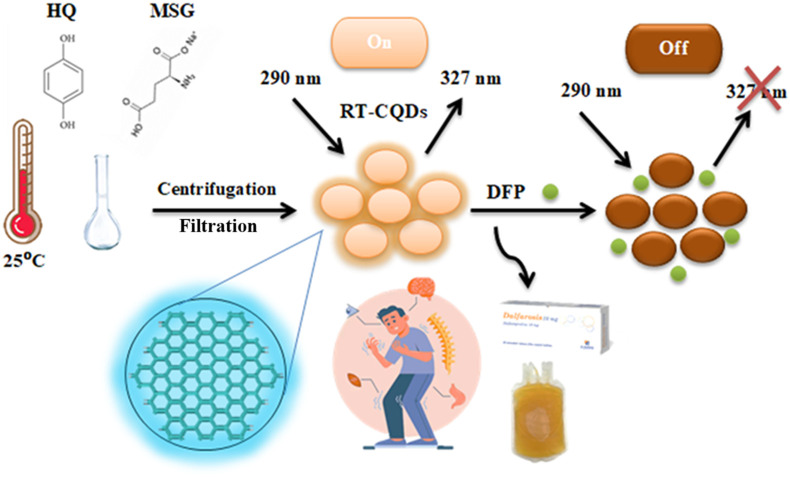

## 1. Introduction

In recent years, carbon nanomaterials (CNMs) have gained increasing attention because of their outstanding optical, electronic, and mechanical characteristics, offering new opportunities for green and sustainable technologies.^1^ Their unique chemical and physical properties make them suitable for many applications, such as nano extraction beds, fluorescence imaging, and fluorescence sensing.^2^ Carbon quantum dots (CQDs) are a promising type of CNMs, well known for their exceptional optical and electrical characteristics, ease of functionalization, and potential biocompatibility.^3^ They are appealing for a wide range of applications like optoelectronics, sensing, and bio-imaging because of their distinctive optical characteristics, which include adjustable emission and fluorescence wavelengths.^4^ Their sensitivity and selectivity make them valuable tools in analytical chemistry.^5,6^ The growing applications of CQDs have led to the development of a number of easy, affordable, and eco-friendly CQD synthesis techniques.^7,8^ In terms of various mechanisms, CQD synthesis can be divided into two categories: “top-down” and “bottom-up”.^9,10^ The “top-down” synthesis techniques involve chemical or physical reduction of big carbon sources to produce miniature CQDs.^11^ Carbon fibers and graphite rods are usually employed as carbon sources in the “top-down” techniques.^12^ In contrast, the “bottom-up” synthesis technique synthesizes CQDs from tiny carbon sources in molecular or ionic forms. CQDs are optimized through synthesis and post-treatment, since their structure and composition determine their various qualities.^13^ CQDs' extraordinary biocompatibility and water solubility are attributed to the abundance of hydrophilic groups on their surfaces, particularly carboxylic and hydroxyl groups.^14,15^ Furthermore, CQDs' chemical inertness enables them to attain widespread acceptance due to their stability and compatibility.^16–20^

Recent developments demonstrated that CQDs may be synthesized at room temperature, offering a more cost-effective and environmentally friendly approach.^21^ Additionally, it is simple to scale up room temperature synthesis, which makes it possible to produce CQDs on a large scale with consistent quality. However, the generated CQDs typically have a very low quantum yield, take a long time to generate, and/or have a hazardous synthetic process that involves high concentrations of strong alkalis like sodium hydroxide, strong acids like nitric acid, or strong oxidizing agents like hydrogen peroxide. Thus, it is crucial to develop a novel and quick room-temperature method for constructing CQDs with a great quantum yield in moderate environments. Many substances, including ethylenediamine,^22^ hydroquinone, and methylglyoxal,^23^ have been employed in different studies for determination of pharmaceutical drugs. In this work, for the first time, mono sodium glutamate (MSG) was utilized with hydroquinone as precursors for room temperature synthesis of CQDs through a very simple reaction. It also has the benefit of not requiring strong alkalis, acids, and oxidants.

Dalfampridine (DFP) is the first FDA-approved medication for improving walking in multiple sclerosis' patients, a disease characterized by nerve dysfunction that causes numbness, weakness, loss of muscular coordination, and issues with speech, vision, and bladder control.^24^ It is an oral potassium channel blocker, chemically named 1,4-dihydropyridin-4-imine. It increases the action potential conduction in demyelinated axons by inhibiting potassium channels. DFP is completely and quickly absorbed orally to achieve relative bioavailability up to 96%, and it is excreted in its unaltered form, primarily from urine (96%). The literature revealed different analytical methods for DFP analysis, including spectrophotometric,^25,26^ spectrofluorimetric,^27^ electrochemical,^28^ and various chromatographic methods.^29,30^ Nevertheless, some disadvantages of most of these methods include the requirement for costly equipment, expensive tests, low detection sensitivity, long preparation and equilibration periods, as well as excessive use of hazardous chemicals, reagents, and organic solvents.^31,32^ These limitations restrict the application of these methods for quality control analysis of DFP. Consequently, it is crucial to develop a new, straightforward, sensitive, economical, as well as widely applicable method for determining DFP in different matrices.^33,34^ Among various analytical techniques, spectrofluorimetry offers many advantages, such as sensitivity, selectivity, quick linear response across a broad concentration range, ease of use, affordability, and adaptability to various matrices.^35–41^

Our goal in this current study is to develop much more affordable, simple, and fast CQDs' formation in order to replace more costly, time-consuming, and labor-intensive CQDs made from costly materials. In this case, hydroquinone and monosodium glutamate (MSG) reacted directly at ambient temperature to effectively produce RT-CQDs. This work intends to construct a straightforward, low-cost, and suitably sensitive fluorescence sensor to quantify DFP in various matrices, such as biological samples and pharmaceutical forms, relied on quenching mechanism.

## 2. Experimental

### 2.1. Chemicals and reagents

Hydroquinone (C_6_H_6_O_2_, El Nasr Pharmaceutical Chemicals Co, Cairo, Egypt) and monosodium glutamate (99.0%; Sigma-Aldrich, Hamburg, Germany) were used to prepare RT-CQDs. Dalfampridine was obtained from Al-Andalus Pharmaceuticals, 6th October City, Egypt. Dalfarosis® tablets (Lot no. # 240054 manufactured by Al-andalous pharmaceutical industries, 6th October City, Egypt) containing 10 mg DFP were gained from a local drugstore. Sodium hydroxide, acetic acid glacial ACS (99.7% Purity; density = 1.05 g mL; Ostim, Ankara, Turkey), orthophosphoric acid (85% purity; density = 1.88 g mL; Ostim, Ankara, Turkey), and boric acid glacial ACS, A.R. (99.5% Purity; density = 1.435 g mL; Ostim, Ankara, Turkey) were utilized to prepare Britton–Robinson buffer (0.04 M, pH 2.0–10.0). Acetonitril, ethanol, and methanol were gotten from Sigma-Aldrich (St. Louis, MO, USA). Human plasma Samples were acquired from Kafrelsheikh University Hospitals (Kafrelsheikh, Egypt) and were stored at −80 °C until desired.

#### 2.1.1. Devices

The emission spectra and fluorescence intensities were recorded on a Cary Eclipse- Agilent Technologies Fluorescence Spectrophotometer (Santa Clara, USA). The UV/VIS spectrophotometer (PG Instrument, UK) was used to obtain the UV/VIS absorption spectra. The prepared RT-CQDs were characterized by several techniques such as, transmission electron microscopy TEM (Tokyo, Japan) for determining the morphology and size, Fourier transform infrared spectrum FTIR (Jasco 6800, Tokyo, Japan) for detecting the surface functional groups, energy-dispersive X-ray spectroscopy attached to a scanning electron microscope EDX (JEOL, JSM-IT100LA, Japan) to determine the elemental contents and types, and zeta potential analyzer for determining the surface electrical charge and stability. All pH values were adjusted by a Jenway pH meter (3510). Freeze dryer (Alpha 1-2 LD plus, Germany) was also used.

### 2.2. Procedures

#### 2.2.1. Synthesis of RT-CQDs

RT-CQDs were synthesized at room temperature using hydroquinone and MSG. In a 10 mL volumetric flask, 0.110 g of hydroquinone and 0.507 g of MSG were combined and completed to the volume by distilled water. After 20 minutes, the mixture was centrifuged at 4000 rpm for 10 minutes. A syringe filter with a pore size of 0.22 µm was utilized to filter the supernatant, which was then stored in a dark container at 4 °C. The production yield was calculated by freeze-drying the RT-CQDs. In contrast to the expected yield of 0.617 g (0.110 + 0.507), the produced powder weighed around 0.45 g. Therefore, our method of synthesizing RT-CQDs at room temperature offers a high output yield of 72.9%. The working solution of RT-CQDs was prepared by adding 300.0 µL of the synthesized RT-CQDs into a 100 mL volumetric flask and completed to the volume by distilled water.

#### 2.2.2. Calculation the fluorescence quantum yield (*Φ*)

With amino pyridine (*Φ* = 0.60) as a standard fluorophore, the synthesized RT-CQDs quantum yield (*Φ* RT-CQDs) was calculated using the single point method.^42^ The absorbance and integrated fluorescence values of amino pyridine in 0.1 M H_2_SO_4_ and RT-CQDs solutions were recorded. The absorbance of both the sample and the standard at the excitation wavelength was maintained below 0.1 to minimize the absorbance effects. The following formula was used to calculate *Φ* RT-CQDs.^43^1
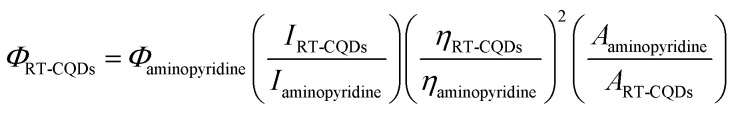
where, *I* stands for the integrated fluorescence intensity. *A* stands for optical absorbance and *η* is the solvent's refractive index. *I*_RT-CQDs_ = 20772, *I*_aminopyridine_ = 34954, *A*_aminopyridine_ = 0.032, and *A*_RT-CQDs_ = 0.03.

#### 2.2.3. Determination of the production yield (PY) of RT-CQDs synthesis

The solution of RT-CQDs was freeze-dried at −65 °C for three days under vacuum to generate solid RT-CQDs. After the prepared solid was weighed, the production yield (PY) was determined. The following eqn (2) was applied to calculate PY.^44^2
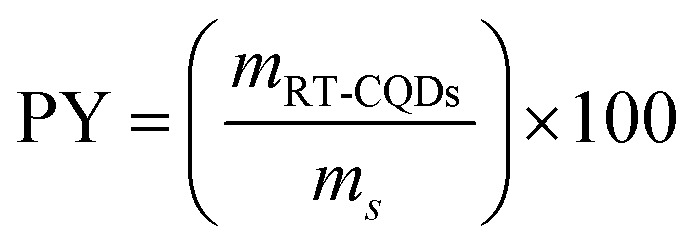
where, *m*_RT-CQDs_ stands for the solid mass of RT-CQDs, and *m*_s_ for the mass of the precursors (hydroquinone and MSG).

#### 2.2.4. Preparation the standard and workable solutions

A precisely weighed quantity (0.01 g) of pure powder was dissolved in 100.0 mL of distilled water to create a freshly-prepared standard solution of DFP (100 µg mL^−1^) in a 100.0 mL volumetric flask. Then, by further diluting with distilled water, workable solutions were prepared in the concentration range of 0.02–14.0 µg mL^−1^.

### 2.3. Procedure for analyzing DFP using RT-CQDs

#### 2.3.1. Construction of the calibration curve

In a set of 5 mL volumetric flasks, 140.0 µL aliquots of RT-CQDs and 400.0 µL of Britton–Robinson buffer (pH 7) were mixed with increasing volumes of the DFP working solutions. To attain homogeneity, the solutions were vigorously mixed after adjusting the final volume of each flask with distilled water. The resultant solutions' fluorescence intensities were determined at *λ*_ex_ 290 nm and *λ*_em_ 327 nm. It was found that the addition of the DFP resulted in a decreasing of the RT-CQD's emission intensity, and the extent of this quenching increased with increasing DFP concentration. A blank test was determined by removing the drug to measure the intrinsic fluorescence intensity of the RT-CQDs (F0). Graphing the DFP concentrations (µg mL^−1^) against fluorescence quenching (Δ*F* = *F*_0_–*F*) produced the calibration curve, which was then used to calculate the regression equation.

#### 2.3.2. Analysis of DFP in pharmaceutical dosage forms

Five Dalfarosis® tablets were precisely weighed and ground to a fine powder. A carefully determined amount of powder, equivalent to 10 mg of DFP, was placed into a 100 mL conical flask. The DFP was extracted by adding 25 mL of methanol and sonicating for around 30 minutes. The flask's final volume was then filled with distilled water, and the resulting solution was filtered through a syringe membrane filter (0.22 µm). The working solution has been prepared by transferring different volumes of the filtrate into a set of 5 mL volumetric flasks. After that, the steps listed in section “2.4.1” were followed. The pre-graphed calibration curve or the regression equation has been used to determine the percentage of drug recovery.

#### 2.3.3. Analysis of DFP in spiked human plasma

In a series of Falcon tubes, various volumes of DFP standard solution aliquots were added to 1 mL of human plasma. A vortex mixer was used to mix the spiked samples for 2.0 minutes. Then, each tube received 140.0 µL of RT-CQDs and 400.0 µL of Britton–Robinson buffer at pH 7. Plasma deproteination was performed by diluting it to 5 mL of methanol. After 2 minutes of vortex mixing, the mixtures were centrifuged for 10 minutes at 10000 rpm. The supernatant was filtered through a 0.22 µm syringe filter. As shown in Section “2.4.1” fluorescence measurements were performed for three DFP concentrations (0.02, 0.4, and 0.6 µg mL^−1^), and the associated regression equation was derived.

#### 2.3.4. Content uniformity testing

The official USP criteria were applied to guarantee that the dosage units' tablets were uniform. The content uniformity test was applied on ten separate tablets utilizing the same method shown in section 2.4.2. Analysis of DFP in pharmaceutical dosage forms. The acceptance value (*A*_V_) was determined utilizing the formula *A*_V_ = |*M* − *X*′| + *k* × *s*,^13^ where *M* represents the reference value, *X*′ is the mean of individual contents, *k* is the acceptability constant, and *s* is the sample standard deviation.^13^ The content uniformity test is passed if the *A*_V_ of ten tablets is lower than or equal to the maximum *A*_V_ allowed by USP (L1; 15 000 unless otherwise specified).^13^

## 3. Results and discussion

### 3.1. Synthesis of RT-CQDs

**Scheme 1:**
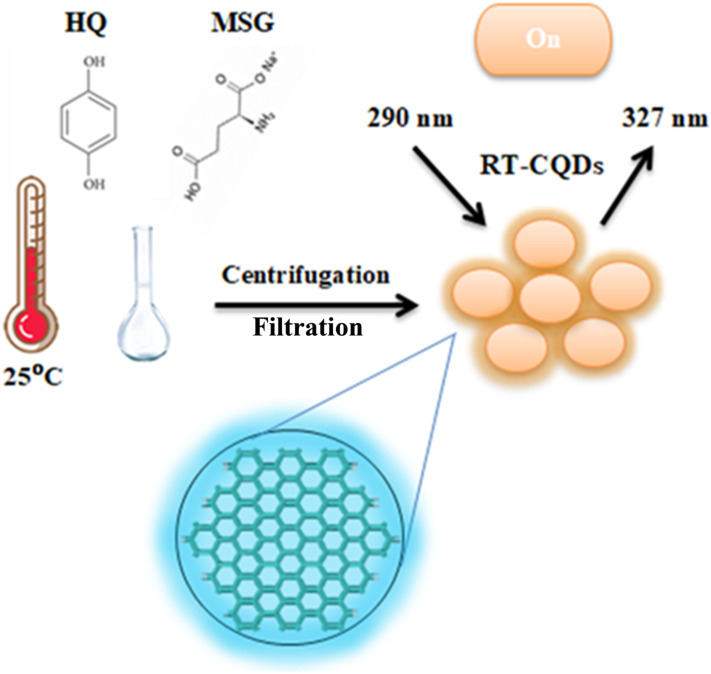
Synthesis of RT-CQDs.

The use of RT-CQDs as a state-of-the-art analytical sensor for the measurement of DFP in pharmaceutical and biological samples was examined in this work. RT-CQDs can offer essential fluorescent nanosensors for detecting a variety of analytes because of their unique optical and electrical properties, including their high photostability, tunable fluorescence, and sufficient quantum yield. As RT-CQDs can selectively bind to drug molecules, the target drugs may be differentiated from several interfering components in different matrices. The low detection limit values demonstrated that RT-CQDs could be a powerful tool for quality control analysis. This study introduces the first spectrofluorimetric method for DFP determination based on RT-CQDs. In the room temperature synthesis of CQDs, hydroquinone served as the carbon precursor and MSG as a carbon source and a nitrogen-atom dopant (Scheme 1). CQDs are made at room temperature utilizing HQ and MSG as precursors, with no distinctive apparatus or oxidant reagents required. Scheme 1 shows the formation of RT-CQDs. HQ readily oxidizes to *p*-benzoquinone in air due to its two active phenolic hydroxyl groups.^45^ As shown in Fig. S1, an oxidative condensation reaction takes place between the C=O of *p*-benzoquinone and the NH_2_ group of MSG.^46^ The heat produced by this reaction accelerates the synthesis of RT-CQDs. Hydroquinone was selected due to its hydroxyl substituents, which offer reactive sites for surface functionalization, and its aromatic benzene ring, which promotes the formation of π–π conjugated domains. These structural characteristics encourage the production of stable, well-dispersible CQDs. Conversely, MSG was added to serve as a heteroatom dopant. Incorporating nitrogen atoms into the CQDs' lattice through surface attachment or carbon atom substitution can improve surface passivation, increase electron density, and inhibit non-radiative recombination pathways. It is well known that these effects improve quantum yield and fluorescence intensity. The photophysical characteristics of the generated RT-CQDs were further optimized by systematically examining various precursor ratios of hydroquinone to the MSG molecule. According to the findings shown in Fig. S2, there was a considerable improvement in quantum yield and enhanced fluorescence emission when the nitrogen content was increased up to a 1 : 3 (w/w) ratio of hydroquinone and MSG, respectively. This is because nitrogen functionalities provide surface energy traps that serve as radiative recombination hotspots. However, too much nitrogen precursor led to partial quenching because of the aggregation or the formation of non-radiative defects. The maximum fluorescence intensity and quantum yield were obtained with the optimal hydroquinone-to-MSG ratio of 1 : 3 (w/w), underscoring the need of precisely adjusting the relative quantities of carbon and nitrogen precursors during synthesis. These results demonstrate that regulating the ratio of nitrogen doping to carbonization (caused by MSG and hydroquinone) is crucial for establishing the optical characteristics of CQDs. Furthermore, compared to traditional high-temperature or hydrothermal procedures, the simple room-temperature synthesis route provides a more environmentally friendly and energy-efficient option, which makes this strategy very appealing for different sensing applications. The quantum yield (*Φ*) of the proposed RT-CQDs was found to be 38.05%, indicating high fluorescence efficiency. This value reflects the effectiveness of the developed RT-CQDs as sensitive nanosensors for the studied analyte, and confirms the successful fluorescence response under the optimized experimental conditions.

### 3.2. Characterization of the RT-CQDs

**Fig. 1:**
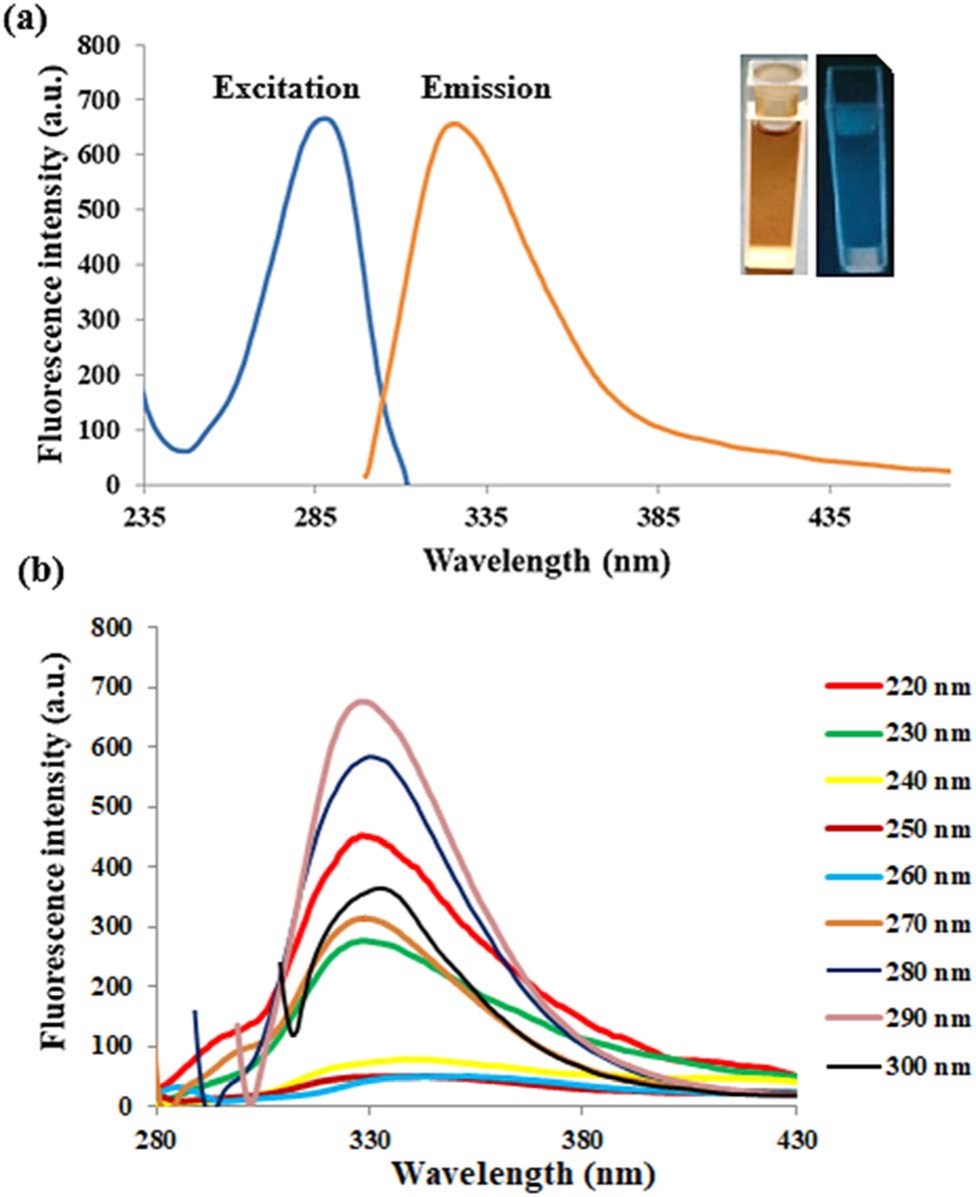
The RT-CQDs' fluorescence excitation and emission spectra of at 290 nm and 327 nm (a) and fluorescence spectra of the RT-CQDs at various excitation wavelengths (220–300 nm) (b).

The structural and optical characteristics of the generated RT-CQDs were investigated. The optical properties of RT-CQDs were further investigated through the utilization of spectrofluorimetry and UV-vis spectrophotometry. As shown in Fig S3, The UV-vis absorption spectrum of RT-CQDs reveals three different absorption peaks at 226, 290 nm, and 348 nm. The π–π* energy transition of the C=C bonds in the aromatic core of the CQDs is represented by the peak at 226 nm,^47^ whereas the *n*–π* transition of the carbonyl/amine functional groups on the CQDs' surface is responsible for the peaks at 290 nm and 348 nm.^48^Fig. 1a also represents that the RT-CQDs exhibit a significant emission peak at 327 nm when excited at 290 nm. The RT-CQDs solution displays an intense blue color when exposed to 290 nm UV light, as demonstrated in the inset photo in Fig. 1a.To examine the characteristic of excitation-dependent emission, the RT-CQDs solution was scanned at excitation wavelengths between 220 and 300 nm (with 10 nm intervals), and the resulting emission responses were determined (Fig. 1b). Changing the excitation wavelength from 220 to 300 nm did not significantly impact the maximum emission wavelength; nevertheless, the fluorescence intensity (FI) enhanced progressively until it reached 290 nm. Furthermore, FI gradually decreases as the excitation wavelength is raised from 290 to 300 nm, which is associated with excitation-dependent down-conversion fluorescence emission.

**Fig. 2:**
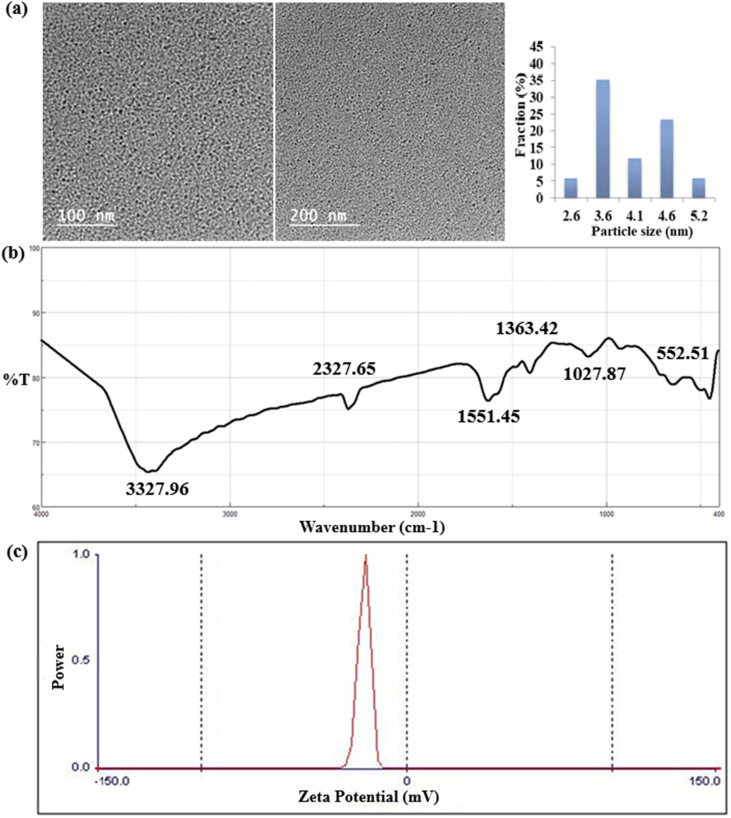
(a) High-resolution TEM (HRTEM) images and particle size distribution, (b) FT-IR spectra, and (c) Zeta potential of the produced RT-CQDs.

The produced RT-CQDs are visible in the HRTEM image as a homogeneous dispersion of spherical nanoparticles over a dark carbon-coated grid. The RT-CQDs' high electron density and nanoscale size are demonstrated by their appearance as distinct, brilliant spots against a contrasting background. The RT-CQDs show a size range predominantly centered around 2.6–5.6 nm with an average size of 3.6. The nanoparticles exhibit little aggregation and good dispersion, indicating their stability and efficient synthesis. As illustrated in Fig. 2a, the morphology and distribution of RT-CQDs highlight their applicability for fluorescence-based applications, including the sensitive anaysis of DFP in pharmaceuticals and plasma samples.

**Fig. 3:**
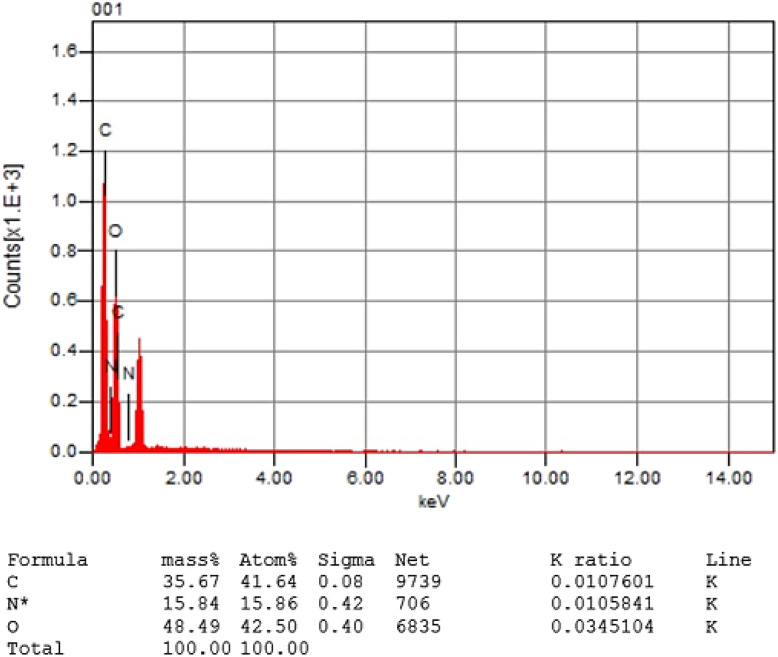
EDX of the prepared RT-CQDs.

According to the infrared spectra (IR), the production of RT-CQDs from hydroquinone and MSG can be vertified by observing key absorption peaks typical of such nanomaterials. The intense and broad band between 3200–3500 cm^−1^ reveals the occurrence of hydroxyl (–N–H–) and (–OH) groups, which is a frequent characteristic on the surface of RT-CQDs due to the oxygen-containing functional groups. The peak at 2327.65 cm^−1^ verifies the existence of (–COO–). The C=C stretching peak at 1551.45 cm^−1^ suggests the potential presence of aromatic compounds, which most likely result from the hydroquinone precursor. The peak at 1363.42 cm^−1^ shows (C–N) stretching which derived from MSG structure.^49^ Furthermore, the peak at 1027.87 and 551.51 cm^−1^ confirms the occurrence of (–C–O) and (–C–H–) stretching (Fig. 2b). A zeta potential analyzer was utilized to evaluate the electron density and surface of RT-CQDs. The zeta potential of the produced RT-CQDs was −27.52 mV, suggesting that they had negatively charged surfaces because of electron-rich doping (a significant amount of oxygen and nitrogen-containing groups) and verifying their stability (Fig. 2c). The quantities and kinds of elements in the RT-CQDs were determined using energy-dispersive X-ray spectroscopy (EDX). Fig. 3 depicts an EDX examination of RT-CQDs, demonstrating that the C and O content are 35.67% and 48.49%, respectively, suggesting that RT-CQDs are largely carbon-based. The N concentration was 15.84%, indicating that RT-CQDs are substantially doped with N. As demonstrated in Fig. S4, the ^1^H NMR spectrum of HQ displayed a sharp singlet at *δ* 6.53 ppm corresponding to the four equivalent aromatic protons, along with a broad signal at *δ* 4.66 ppm assigned to the hydroxyl protons, reflecting the molecule's high para-symmetry and the absence of aliphatic protons. In contrast, the spectrum of MSG showed no aromatic signals, featuring instead a characteristic α-proton resonance at *δ* ∼4.0 ppm. The peak at *δ* 3.8 ppm is assigned to protons adjacent to nitrogen atom, indicating the presence of heteroatom-containing functional groups. The two sets of multiplets between *δ* 1.8–2.5 ppm corresponding to the β- and γ-methylene protons, consistent with its fully aliphatic structure (Fig. S5). The ^1^H NMR of the synthesized carbon quantum dots (CQDs) revealed broad signals in both the aromatic region (∼6.8–7.0 ppm) and the aliphatic regions (∼2.0–4.0 ppm), indicating the formation of surface-functionalized carbon domains (Fig. S6). The data suggest that the interaction between HQ and MSG under room temperature aqueous conditions is better described as oxidative condensation leading to surface functionalization of the resulting CQDs. Notably, no downfield shifts or signals characteristic of ester protons were observed, supporting that the reaction proceeded primarily through oxidative condensation rather than classical esterification.

### 3.3. Mechanism of RT-CQDs sensing system

**Fig. 4:**
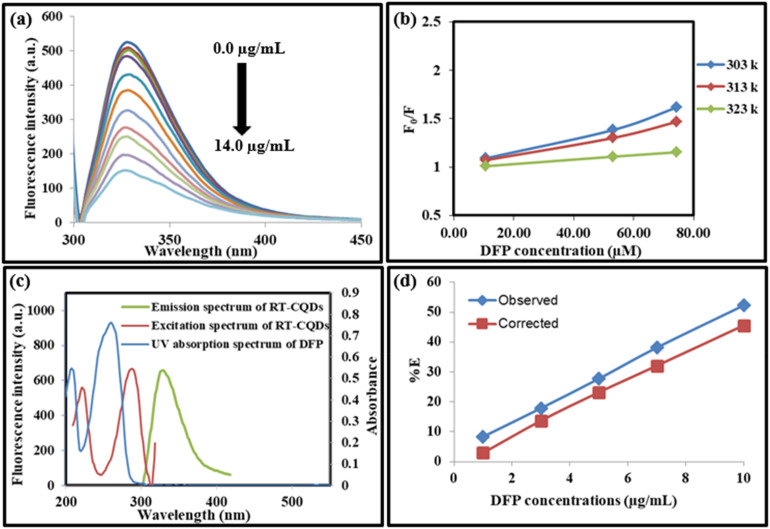
(a) The RT-CQDs' fluorescence emission spectra after adding different concentrations of DFP (from top to bottom: 0, 0.02, 0.2, 1.0, 3.0, 5.0, 7.0, 9.0, 10.0, 12.0, 14.0 µg mL^−1^), (b) Stern–Volmer plots for fluorescence quenching of RT-CQDs by DFP (10.63, 53.13, 74.38 µM) at three various temperature (303, 313, and 323 K), (c) A co-plot demonstrating the overlap between the UV-vis absorption spectrum of DFP and the fluorescence excitation spectrum of RT-CQDs, (d) % *E* of observed and corrected fluorescence of RT-CQDs after adding different concentrations of DFP.

Some of the probable quenching mechanisms involve static quenching mechanism, dynamic quenching mechanism, and the inner filter effect (IFE). Static quenching results from the formation of a non-fluorescent ground state complex between the quencher and fluorophore, whereas collisional quenching, also referred to as dynamic quenching, occurs when an excited fluorophore returns to its ground state as a result of interactions or collisions with a quencher. *K*_sv_ values rise with rising temperature in dynamic quenching, but decrease with increasing temperature in static quenching. As DFP concentrations increase from 0.02 to 14.0 µg mL^−1^, the produced RT-CQDs' intense fluorescence is turned off (Fig. 4a). As a starting point to investigate the mechanism of the fluorescence quenching, the Stern–Volmer equation was used at three distinct temperatures (303, 313, and 323 K) as follows:3
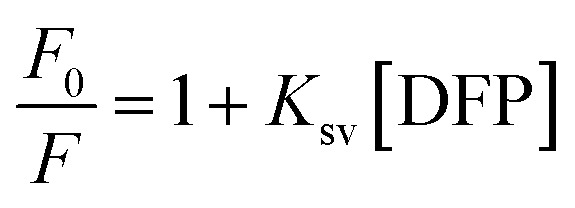
*K*_sv_ represents the Stern Volmer quenching constant, while *F*_0_ and *F* stand for the fluorescence intensities of RT-CQDs prior to and following the mentioned drug addition, respectively. Fig. 4b displays plots of *F*_0_/*F versus* [DFP] at different temperatures. The computed values of *K*_sv_ at 303, 313, and 323 K were 8.044 × 10^3^, 6.093 × 10^3^, and 2.269 × 10^3^ L mol^−1^, respectively. The *K*_sv_ results decreased as the temperature rose, suggesting that the DFP-induced emission quenching mechanism of RT-CQDs followed a static quenching mechanism in which the complexation occurred between the quencher (DFP) and the fluorophore (RT-CQDs). Furthermore, *k*_q_ values were computed using the obtained *K*_SV_ values in the equation described below.

4 *K*_q_ = *K*_sv_/*τ*_0_

where; *τ*_0_ is the average lifetime of RT-CQDs (10^−8^*s*) and *K*_q_ is the quenching rate constant. Static quenching was further confirmed by the computed *K*_q_ values, which were 8.044 × 10^11^, 6.093 × 10^11^, and 2.269 × 10^11^ L mol^−1^ S^−1^ at (303, 313, and 323 K), respectively. The calculated *K*_q_ values were significantly higher than the maximum diffusion-controlled rate constant (2.0 × 10^10^ L mol^−1^ S^−1^), confirming that the quenching mechanism follows a static pathway *via* ground–state complex formation rather than a dynamic process. Additionally, it was noted that the DFP’ UV spectrum nearly overlapped with the excitation spectrum of RT-CQDs (Fig. 4c). This suggests the possibility of the IFE mechanism, in which DFP partially absorbs the excitation light, lowering the RT-CQDs' excitation and, consequently, the detected fluorescence. The fluorescence intensity of RT-CQDs was corrected by applying the following equation after different concentrations of DFP were added.5
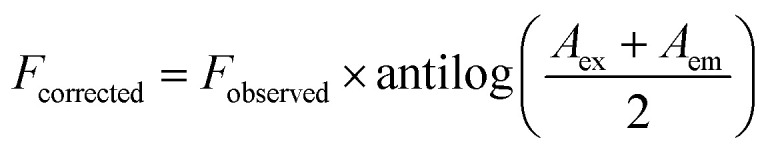
where *F*_observed_ represents the fluorescence intensity as it was observed, and *F*_corrected_ represents the fluorescence intensity after the inner filter effect is subtracted. The drug's absorbance values at 290 nm and 327 nm were indicated by the *A*_ex_ and *A*_em_, respectively. The fluorescence suppression efficiency (% *E*) for both *F*_observed_ and *F*_corrected_ was then computed using the given equation.6
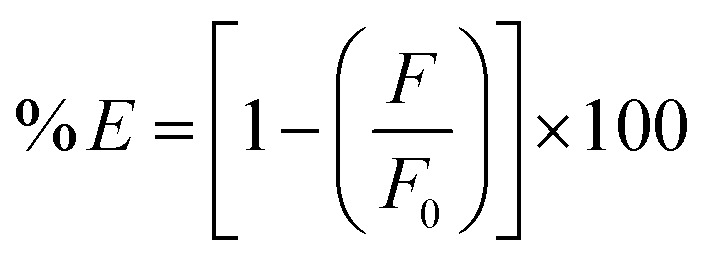


According to the findings demonstrated in Fig. 4d, it was observed that IFE contributed about 10% of the maximum fluorescence quenching, making it a secondary mechanism, with static quenching being the predominant mechanism. Static quenching decreased the quantity of RT-CQDs through complexation, whereas IFE diminished the emitted light.

### 3.4. Method optimization

In order to improve the DFP determination performance, the effects of RT-CQDs volume, buffer pH, buffer volume, diluting solvent, and incubation time on the fluorescence intensity of RT-CQDs with and without DFP addition were studied. Fig. S7a demonstrated the fluorescence quenching intensity (*F*_0_ – *F*, where *F*_0_ and *F* represent the emission intensity before and after the DFP addition) at varying volumes of the RT-CQDs solution with the same DFP concentration of (7.0 µg mL^−1^). The fluorescence quenching intensity reached its maximum value at a volume of 140.0 µL of RT-CQDs and rapidly decreased above this volume. Thus, the optimum volume of RT-CQDs was selected to be 140.0 µL. The influence of pH on the fluorescence quenching of RT-CQDs by DFP was investigated using Britton Robinson buffer (BRB) in the pH range of 2–10. As shown in Fig. S7b, it was noted that BRB with a pH of 7 increased DFP's ability to quench RT-CQDs fluorescence. Accordingly, several buffer volumes between 100.0 and 600.0 µL were used to investigate the influence of BRB buffer volume (pH 7), and 400.0 µL was observed to be the ideal volume (Fig. S7c). The influences of different diluting solvents, such as methanol, ethanol, acetonitrile, and distilled water were studied. As shown in Fig. S7d, the highest difference between *F*_0_ and *F* was observed when the distilled water was used. Fig. S7e depicts the influence of various incubation durations on the Δ*F*. The experimental findings showed that the Δ*F* remained steady from 0 to 60 minutes, proving the instantaneous reaction between CQDs and DFP.

### 3.5. Method validation

The developed approach was validated according to the ICH recommendations. The validation study's findings demonstrate the effectiveness and suitability of the proposed approach, ascertaining its practical application in biological and pharmaceutical analysis. Under ideal conditions, the developed method showed outstanding linearity across the concentration range of 0.02–14.0 µg mL^−1^ with a correlation coefficient value (*r*^2^) of 0.9998 (Table 1). The calibration curve was produced by graphing the relative fluorescence intensity (*F*_0_ – *F*) *versus* DFP concentration, resulting in the linear regression equation of *y* = 25.62*x* + 17.91 (Fig. S8). The method's sensitivity was evaluated by calculating the detection and quantification limits. The following equations could be used to measure the limit of detection (LOD) and limit of quantification (LOQ) : LOD = 3.3*x*/*b*, LOQ = 10*x*/*b*, where *b* is the slope and *x* represents the blank's standard deviation. The developed method was able to detect and quantify DFP at trace levels, as evidenced by the computed LOD and LOQ values of 0.005 µg mL^−1^ and 0.016 µg mL^−1^, respectively. The method's accuracy was assessed through recovery trials at three concentration levels, demonstrating mean recovery of 100.51 ± 0.62%, proving excellent accuracy (Table S1). Both intra-day repeatability and inter-day intermediate precision were used to assess the method's precision (Table S2). According to the relative standard deviation (RSD%), the repeatability was 0.89.The identical concentration levels were analyzed on three consecutive days, producing an RSD of 0.91. These low RSD values demonstrate the great precision of the suggested approach. As shown in Table S3, the analytical approach's robustness was tested by making slight changes in important experimental factors. Minor variations in buffer pH (±0.5 units), buffer volume ((±5.0 µL), and RT-CQDs' volume (±5.0 µL) showed no significant influence on the fluorescence intensity, with recovery values of 99.52 ± 0.44%, 101.06 ± 0.73%, and 100.75 ± 0.63%, respectively. Furthermore, solution stability tests were carried out to assure analytical reliability under varying conditions. When maintained in amber bottles at 4 °C, the stock solution of RT-CQDs exhibited high stability, with less than 2% fluorescence response change over 3 months. The reaction between RT-CQDs and DFP drug demonstrated exceptional photostability, with negligible effect (<2% intensity loss) throughout 60 minutes exposure durations, ensuring accurate results during lengthy analytical procedures. These findings demonstrate that the RT-CQDs' sensing capabilities are reliable and repeatable for everyday analytical applications. The suggested fluorescent probe's selectivity was evaluated utilizing 1000.0 mM of a variety of known inorganic ions (Na^+^, K^+^, Ca^2+^, pb^2+^, Ba^2+^) and possible interferents, such as lactose, mannitol, maltose, dextrin, carboxy methyl cellulose (CMC), and magnesium stearate (Fig. S9). Across all examined species, only DFP caused a significant decrease in fluorescence signal, whereas other chemicals caused minor interference. These findings show the probe's high specificity for DFP, highlighting its potential for selective detection even in complicated pharmacological or biological matrices.

**Table 1:** Analytical performance parameters for DFP determination by the proposed method

Parameter	DFP
Excitation wavelength (nm)	290
Emission wavelength (nm)	327
Concentration range (µg mL^−1^)	0.02–14.0
Slope (*b*)	25.62
Intercept (*a*)	17.91
Correlation coefficient (*r*)	0.9998
S.D. of residuals (*S*_*y*/*x*_)	1.98
S.D. of intercept (Sa)	1.01
S.D. of slope (*S*_b_)	0.13
S.D. of blank (x)	0.04
LOD (µg mL^−1^)	0.005
LOQ (µg mL^−1^)	0.016

### 3.6. Application of the proposed approach

#### 3.6.1. Determination of DFP in Dalfarosis® tablets

The current study was used to quantitatively analyze the DFP drug in its pharmaceutical tablets. The medication concentrations in tablets were ascertained utilizing the corresponding regression equations. As shown in Table 2, the average percentage recoveries of DFP concentrations in its tablet formulations were satisfactory. The results of the developed approach demonstrated a high% recovery of 99.89 and low RSD of 1.33, suggesting that the proposed method is precise and accurate.

**Table 2:** Determination of DFP in pharmaceutical formulations (Dalfarosis® tablets) and spiked human plasma

Sample	Conc. Taken (µg mL^−1^)	Mean conc. Found^a^ (µg mL^−1^)	% recovery found^b^	Mean% recovery ± SD
Dalfarosis® tablets	3.0	2.95	98.37	99.89 ± 1.32
	7.0	7.06	100.79	
	10.0	10.14	100.49	
Spiked human plasma	0.02	0.019	97.70	99.29 ± 1.40
	0.4	0.401	100.34	
	0.6	0.599	99.85	

a Average of triplicate determinations.

b Mean percentage recovery found of three concentrations.

#### 3.6.2. Determination of DFP in spiked human plasma

The developed study's great sensitivity and selectivity enabled the determination of DFP in spiked human plasma. Taking 10.0 mg of DFP results in a mean plasma concentration of 0.025 µg mL^−1^.^50^ When DFP concentrations in µg mL^−1^ were plotted *versus* fluorescence quenching (*F*_0_ – *F*), a linear relationship was observed in plasma samples that had been spiked with DFP. A statistical examination of the data revealed that the mean percentage recoveries ± SD of DFP in plasma samples were 99.29 ± 1.40 (Table 2). The linear regression analysis is described by the following equation: *y* = 42.27*x* + 17.46.

#### 3.6.3. Content uniformity testing of Dalfarosis® tablets

DFP concentrations in its low-dose tablets can be determined due to the developed method's exceptional sensitivity and selectivity. The same method described in the section on “Assay of DFP in Dalfarosis® tablets ” was used to analyze ten tablets of DFP separately. The content uniformity of tablet was assessed using the official USP guidelines (The United States Pharmacopeia 36 NF 31).^51^ As shown in Table 3, the computed acceptance value (*A*_V_) was lower than the USP-recommended maximum permissible acceptance value (L1).^52^

**Table 3:** Content uniformity test results for DFP tablets using the developed approach

Parameters	Tablets no.	% Of the labeled claim
		DFP
		Dalfarosis® (10.0 mg per tablet)
		% Recovery
	1	101.88
	2	100.34
	3	100.34
	4	101.71
	5	101.49
	6	101.59
	7	101.64
	8	100.90
	9	101.42
	10	99.84
Mean		101.12
S.D		0.71
% RSD		0.70
% Error		0.22
Acceptance value (AV)		2.82
Maximum allowed AV (L1)		15.00

### 3.7. Assessment of the method's greenness

**Fig. 5:**
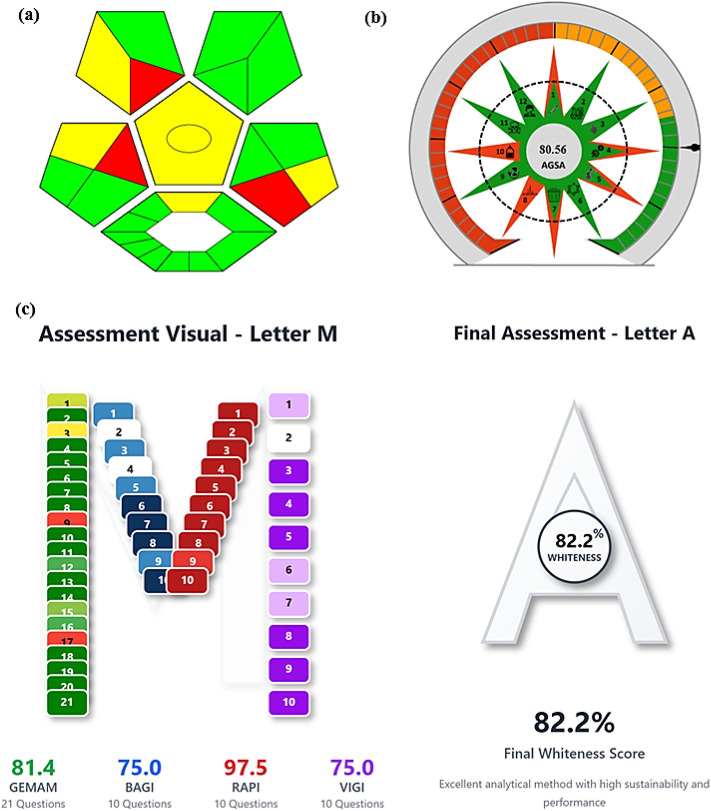
Greenness and whiteness assessment of the developed method using ComplexGAPI (a), AGSA (b), and MA (c) metrics.

Resolving global environmental problems and controlling pollution levels require green chemistry. Consequently, it becomes crucial to create and evaluate green analytical methods that exclude hazardous compounds, employ renewable green substances, and use the least amount of energy.^53–55^ Various metrics were used to assess the greenness of the developed approach, such as the complementary green analytical procedure index (ComplexGAPI)^56^ and the Analytical Green Star Area (AGSA) calculator.^57^ ComplexGAPI software is especially designed to evaluate the greenness of analytical methods that involve materials prepared prior to the analytical step, such as RT-CQDs in the proposed method. In ComplexGAPI, the top pentagons reflect the green characteristics of the analytical method including procedures, reagents, and equipment, taking into account numerous parameters such as amount, solvent and reagent safety, health effect, waste formation, energy consumption, and nature of the method. In addition, the hexagon at the bottom represents the amount of greenness in the synthesis stage (Fig. 5a). It entails an assessment of the circumstances, health and safety hazards, workup, and purification. For low, medium, and high environmental dangers, the green, yellow, and red colors are used. The generated pictogram (Fig. 5a) demonstrated that both processes utilized in the preparation of RT-CQDs and the analytical method meet several green requirements. In addition, the AGSA technique was used to evaluate the suggested method's greenness. This tool measures the greenness of the method by evaluating the toxicity, amounts, automation, produced waste, as well as spent energy. The obtained score of AGSA is 80.56, confirming the greenness of the developed method (Fig. 5b). The results of greenness assessment tools confirm the greenness of the proposed approach. The synthesis of RT-CQDs was easily accomplished under normal conditions that are safe for the environment and humans. When compared to prior hydrothermal and microwave approaches, the new methodology is less costly, requires less energy, and consumes less time. Furthermore, it is based on the synthesis of RT-CQDs from available substances in distilled water, making it more environmentally friendly by eliminating harmful solvents. The proposed method includes various green properties that help to apply the sustainability idea to analytical chemistry laboratories.

### 3.8. Assessment of the method's greenness, practicality, novelty, and performance

In contrast to ComplexGAPI, and AGSA, which primarily estimate an analytical method's greenness, the recently developed Multi-Color Assessment (MA) tool assesses greenness, practicality, novelty, and performance of an analytical method. The MA Tool, a comprehensive online platform that combines four pre-existing assessment tools into one assessment index. Green Evaluation Metric for Analytical Methods (GEMAM), Blueness evaluation (BAGI), Redness Analytical Performance Index (RAPI), and Violet evaluation (VIGI) are all included in the MA Tool through a structured 51-question assessment process. The platform evaluates the technical innovation, operational viability, environmental impact, and analytical performance, assigns a distinct score to each parameter, and then calculates a whiteness score that indicates the method's overall sustainability.^58^Fig. 5c illustrates that the suggested approach obtained an outstanding whiteness score of 82.2.

### 3.9. Comparison of the proposed method with the different published methods for DFP analysis and synthesis of RT-CQDs

The developed approach is significantly more sensitive than reported methods^25–30^ (Table 4). The spectrophotometric methods lack the necessary sensitivity and selectivity.^25,26^ The published spectrofluorimetric method requires derivatization reaction with fluorescamine in borate buffer (pH 8.5) for 5 min.^27^ Electrochemical techniques need many tools and chemical compounds, including glass Petri dishes, polyvinyl chloride (PVC), phosphotungstic acid (PT), sodium tetraphenylborate (TPB), sodium phosphomolybdate (PM), dioctyl phthalate (DOP), and Tetrahydrofuran (THF),^28^ some of which are poisonous. Since their required equipment are expensive, sophisticated, need a lot of preparatory processes, and have a low sample throughput, their availability in quality control labs may be limited.^28^ The chromatographic processes need expensive organic solvents, generate a lot of waste, require highly experienced analysts, take a long time to prepare the column, and need sophisticated equipment.^29,30^ Our method is the first spectrofluorimetric method based on RT-CQDs for DFP detection with greater sensitivity and selectivity. As a declaration of significance, the proposed method is significantly simpler, safer, and more sensitive, with a better regression line shown by a higher *r* value. These properties make the current methodology more practical and applicable in quality control analysis than the majority of previously described approaches. Moreover, compared to several previously published CQD-based sensors synthesized through hydrothermal or microwave-assisted methods, the present RT-CQDs were prepared at room temperature without the need for elevated temperature, high pressure, or intensive energy input. Hydrothermal approaches typically require temperatures above 160 °C and prolonged reaction times extending to several hours.^59^ Although microwave-assisted techniques reduce reaction time, they still involve high-energy irradiation and specialized equipment.^60^ Such conditions certainly enhance energy consumption and may limit the overall sustainability of the procedure. Additionally, few CQD synthesis methods reported in the literature have been carried out at room temperature and successfully applied in analytical sensing. However, most of these approaches have a very low quantum yield, take a long time to generate, and/or have a hazardous synthetic process that involves high concentrations of strong alkalis like sodium hydroxide, strong acids like nitric acid, or strong oxidizing agents like hydrogen peroxide.^22,23,61,62^ From a green chemistry perspective, the proposed synthesis route demonstrates clear advantages in terms of lower energy demand, operational simplicity, and safer reaction conditions. Therefore, beyond comparable analytical performance, the current system provides a significant advantage in terms of greenness, simplicity, and reduced energy consumption, making it more suitable for sustainable analytical applications. Table 5 demonstrated a comparison of the reported CQD based sensors and the developed room-temperature approach.

**Table 4:** Comparison of different reported methods for DFP determination by the proposed method^a^

Technique	Conditions	Linearity (µg mL^−1^)	LOD (µg mL^−1^)	RSD (%)	Sample	Ref.
UV-spectrophotometry	Direct UV determination of DFP at *λ*_max_ = 262 nm	0.2–12.0	0.011	1.38	Pharmaceutical dosage forms and bulk	25
UV-spectrophotometry	Direct UV determination of DFP at *λ*_max_ = 244 nm using g 0.1 N NaOH as a diluting solvent	5.0–25.0	0.125	0.26	Synthetic mixture	26
Spectrofluorimetry	Derivatization by fluorescamine in borate buffer (pH 8.5)	0.02–0.1	0.01	1.86	Synthetic mixture and spiked human plasma	27
HPLC	HPLC determination of DFP by using mobile phase of methanol: acetonitrile (50 : 50v/v) at flow rate of 1.0 ml min^−1^	5.0–25.0	0.54	0.37	Synthetic mixture	26
Electrochemistry	Potentiometric determination of DFP by using PVC membrane	1.88–941.1	1.67	—	Fampyra® tablets	28
HPLC	HPLC determination of DFP by using mobile phase orthophosphoric acid: acetonitrile (60 : 40 v/v) at a flow rate of 0.5 mL min^−1^	25.0–75.0	0.711	0.55	Pharmaceutical dosage forms and bulk	29
HPLC	Precolumn derivatized of DFP with (NBD-Cl) and its HPLC-flu detection at 365 nm	0.01 × 10^−3^–20.0 × 10^−3^	0.003 × 10^−3^	2.45	Human plasma	30
Spectrofluorimetry	By using RT-CQDs for DFP determination	0.02–14.0	0.005	0.89	Pharmaceutical tablets and spiked human plasma	This work

a HPLC: high performance liquid chromatography, PVC: polyvinyl chloride, NBD-Cl: 7-chloro-4-nitrobenzofurazan.

**Table 5:** Comparison of the reported CQD based sensors and the developed room-temperature approach

CQDs	Starting materials	Technique	Synthesis time	QY (%)	Analyte	Ref.
N-doped CQDs	Ammonium citrate and ammonium thiocyanate	Hydrothermal	6 h	61	Clioquinol	59
N,S-CQDs	Thiosemicarbazide and citric acid	Microwave	3 min	6.7	Nitroimidazole antimicrobials	60
RT-CQDs	1,2-Naphthoquinone sulphonate and ethylenediamine	Room temperature	3 h	34.1	Vincristine	22
RT-CQDs	Methylglyoxal and ethylenediamine	Room temperature	20 min	10.02	Fenofibrate	23
RT-CQDs	Sodium 1,2-naphthoquinone-4-sulfonate (Folin's) with ethanolamine	Room temperature	2 min	26.6	Nitroxinil	61
RT-CQDs	HQ, ethylenediamine, and hydrogen peroxide	Room temperature	2 min	36.4	Hydroxocobalamin	62
RT-CQDs	HQ and MSG	Room temperature	10 min	38.05	Dalfampridine	This work

## 4. Conclusion

In this work, a novel and efficient method for producing highly luminescent RT-CQDs at ambient temperature was developed. This approach uses HQ and MSG to synthesize RT-CQDs, with no heating or energy consuming. DFP, a non-fluorescent drug, quantified the fluorescent intensity of RT-CQDs, permitting the development of the first fluorescence nanoprobe for DFP analysis. Both the static quenching and inner filter effect (IFE) mechanisms were responsible for the fluorescence quenching caused by DFP addition. The experimental conditions were carefully adjusted to improve sensitivity. Under these circumstances, the probe demonstrated a broad linearity range from 0.02 to 14.0 µg mL^−1^, with a remarkable LOD of 0.005 µg mL^−1^ and a great quantum yield of 38.05. Interestingly, the probe showed excellent selectivity against known interferents and was effectively employed for DFP determination in pharmaceutical dosage forms, and spiked human samples, demonstrating its practical usefulness in real-world analysis. By eliminating energy consuming, great temperature, and toxic solvents during the preparation of CQDs, the method accomplished exceptional greenness, whiteness, and blueness profiles based on the newly published assessment tools. This opens a new way for cost-effective, safe, and environmentally friendly CQD production.

## Conflicts of interest

The authors declare that they have no known competing financial interests or personal relationships that could have appeared to influence the work reported in this paper.

## Supplementary Material

RA-016-D5RA09768A-s001

## Data Availability

The datasets generated and/or analyzed during the current study are available from the corresponding author on reasonable request. Supplementary information (SI) is available. See DOI: https://doi.org/10.1039/d5ra09768a.
