# Supplementary material for: A novel room-temperature CQD fluorescent nanosensor for the first derivatization-free spectrofluorimetric determination of dalfampridine: application to biological fluids and content uniformity testing
Source: RSC Adv. 2026 Mar 16;16(16):14406–19. doi: 10.1039/d5ra09768a (PMC12991200; doi:10.1039/d5ra09768a)
Supplement: RA-016-D5RA09768A-s001 [file RA-016-D5RA09768A-s001.pdf]

Supplementary Information (SI) for RSC Advances.
This journal is © The Royal Society of Chemistry 2026

Supplementary material

A Novel Room-Temperature CQD Fluorescent Nanosensor for the First Derivatization-Free Spectrofluorimetric Determination of Dalfampridine: Application to Biological Fluids and Content Uniformity Testing

Rehab H. Elattar[a*], Manal A. Alossaimi [b], Ahmed Emad F. Abbas[c], Anmar Anwar Khan[d], Bodour S. Rajab[d], Mazen M. Ghaith[d], Ahmed H Qasem[d], Galal Magdy[e,f*]

[a] Pharmaceutical Analytical Chemistry Department, Faculty of Pharmacy, Suez Canal University, Ismailia, Egypt.

[b] Pharmaceutical Chemistry Department, College of Pharmacy, Prince Sattam bin Abdulaziz University, Al-Kharj 11942, Saudi Arabia.

[c] Analytical Chemistry Department, Faculty of Pharmacy, October 6 University, 6 October City, Giza 12585, Egypt.

[d] Department of Clinical Laboratory Sciences, Faculty of Applied Medical Sciences, Umm Al-Qura University, Makkah 21955, Saudi Arabia.

[e] Pharmaceutical Analytical Chemistry Department, Faculty of Pharmacy, Kafrelsheikh University, Kafrelsheikh, 33511, Egypt.

[f] Department of Pharmaceutical Analytical Chemistry, Faculty of Pharmacy, Mansoura National University, Gamasa, 7731168, Egypt.

***Correspondence:** Galal Magdy: galal_magdy@pharm.kfs.edu.eg, Rehab H. Elattar: rehabhamdy216@gmail.com.

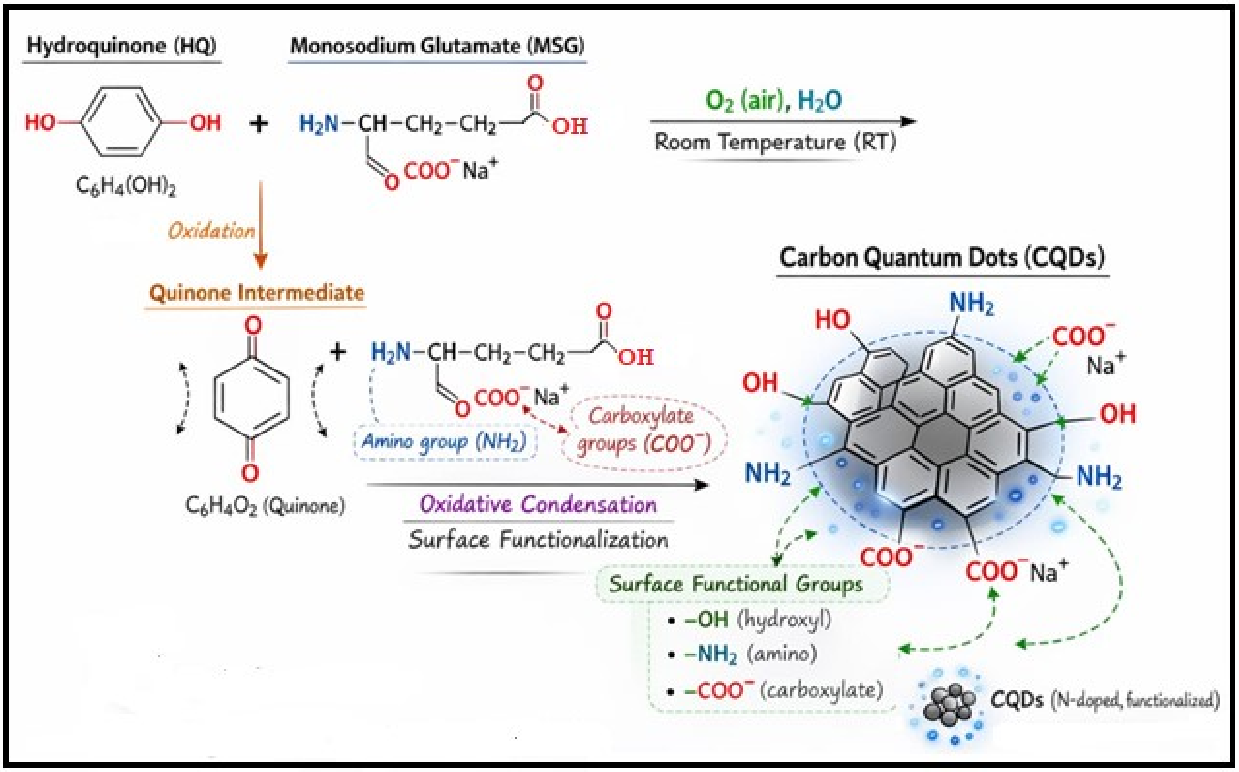

Fig. S1 Schematic representation of the proposed reaction mechanism between HQ and MSG to form RT-CQDs.

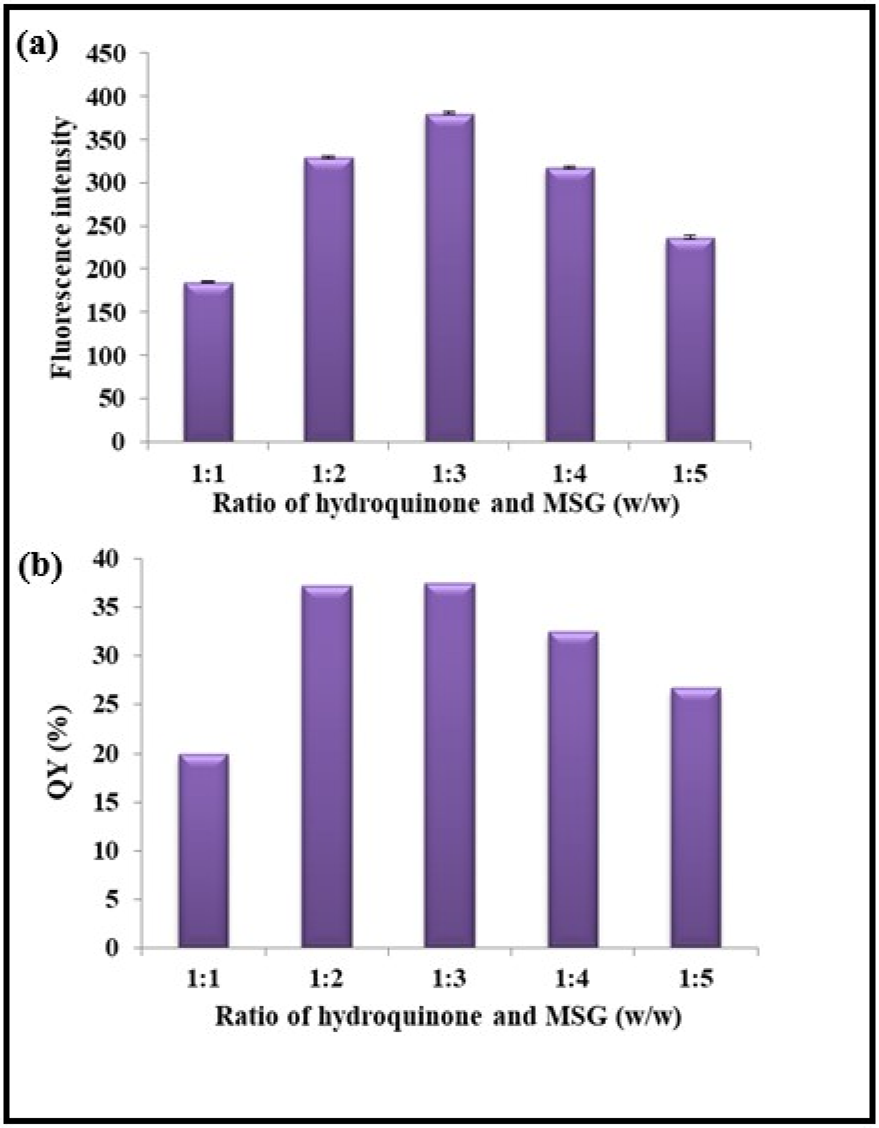

Fig. S2 Effect of different ratios (w/w) of hydroquinone and MSG on the fluorescence intensity (a) and quantum yield of RT-CQDs (b).

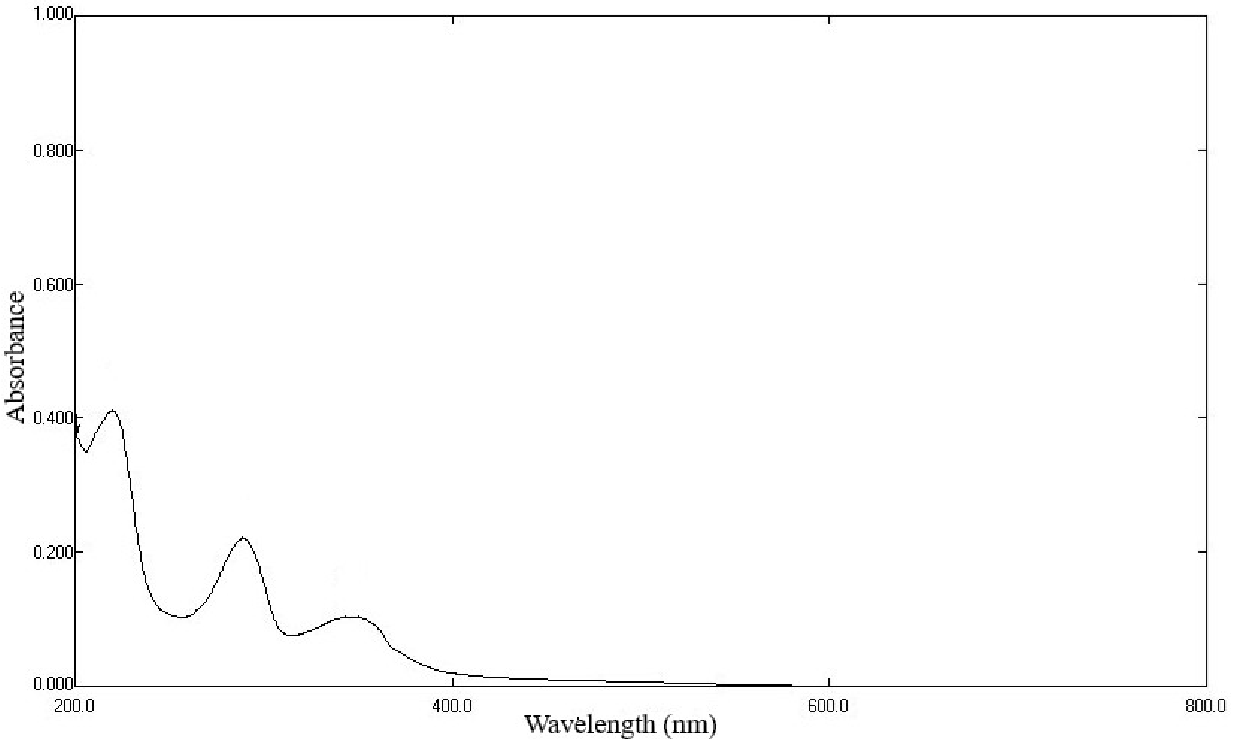

Fig. S3 UV spectrum of the RT-CQDs.

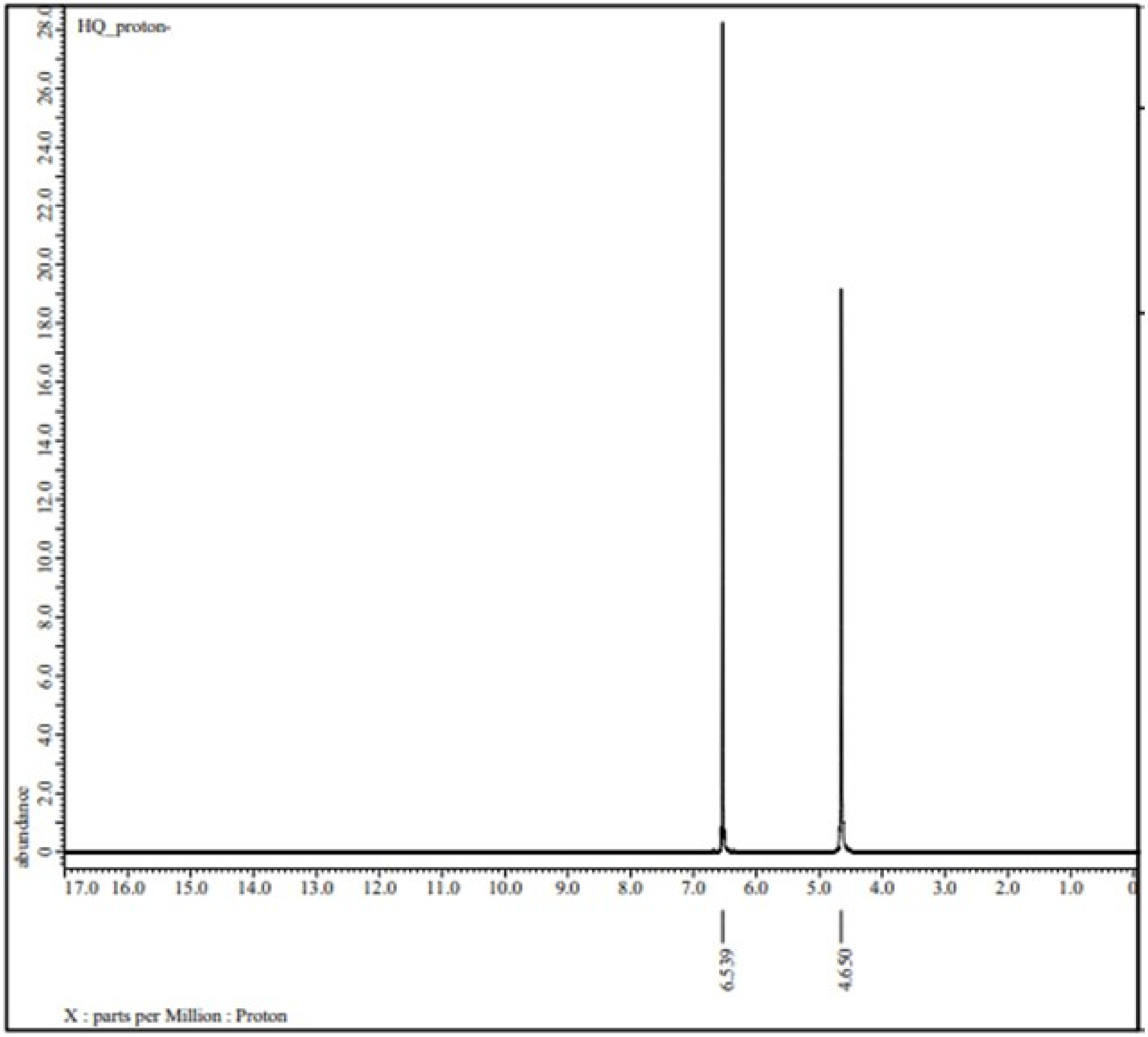

Fig. S4 NMR spectrum of HQ.

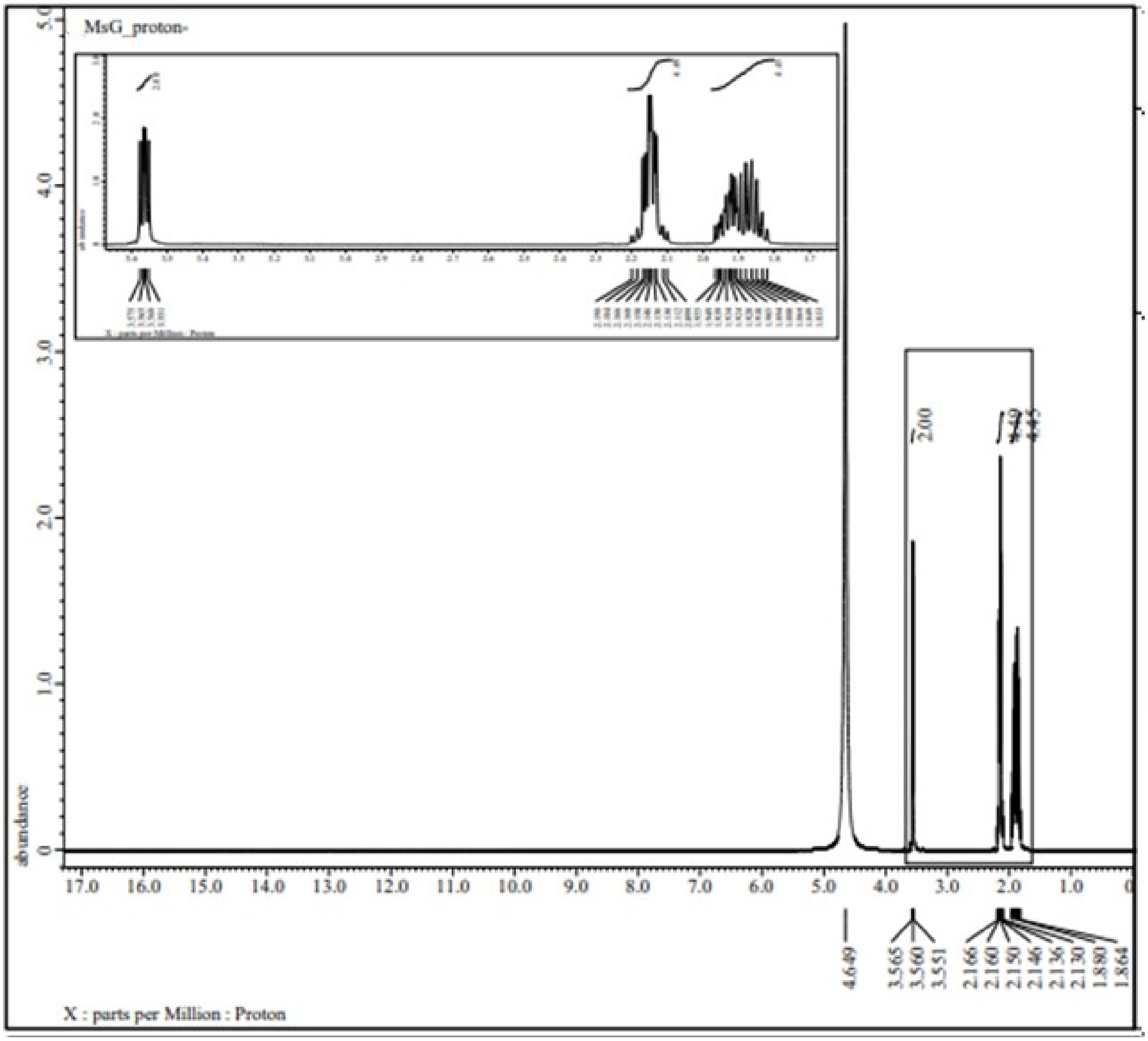

Fig. S5 NMR spectrum of MSG.

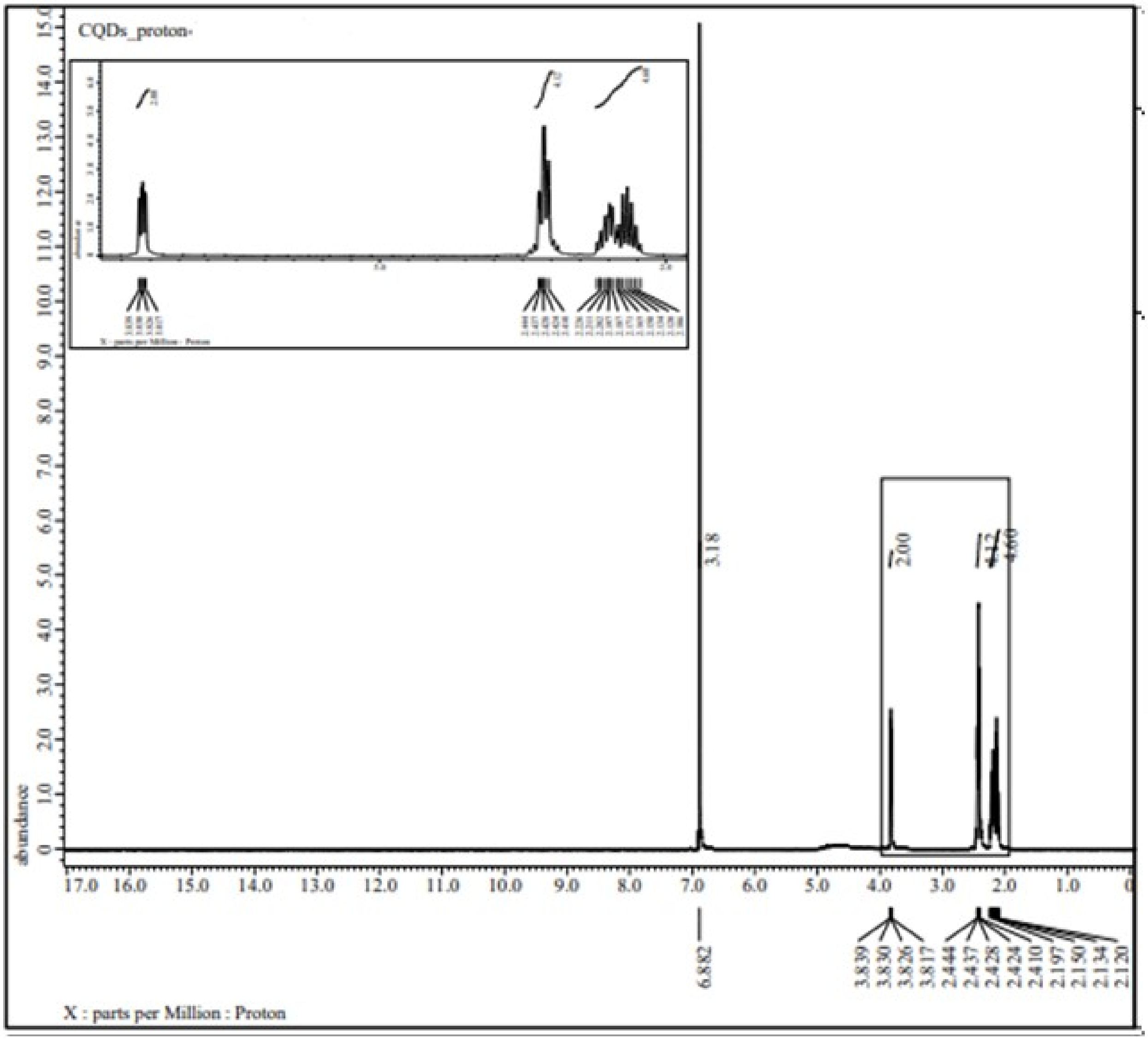

Fig. S6 NMR spectrum of RT-CQDs.

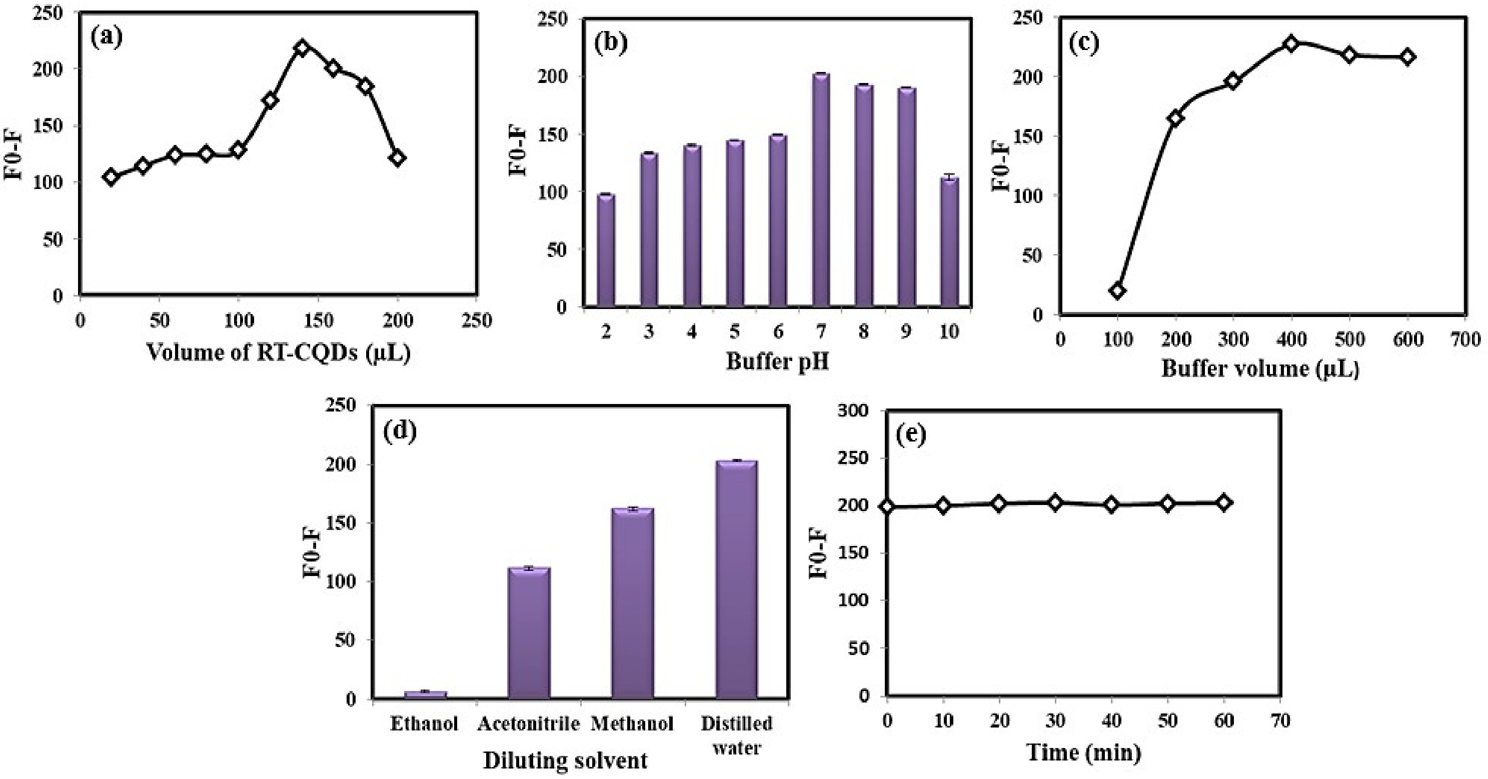

Fig. S7. Effect of the RT-CQDs' volume (a), buffer pH (b), the buffer volume (c), Type of diluting solvent (d), and the incubation time (e) on the fluorescence quenching (F0-F) determined at 327 nm.

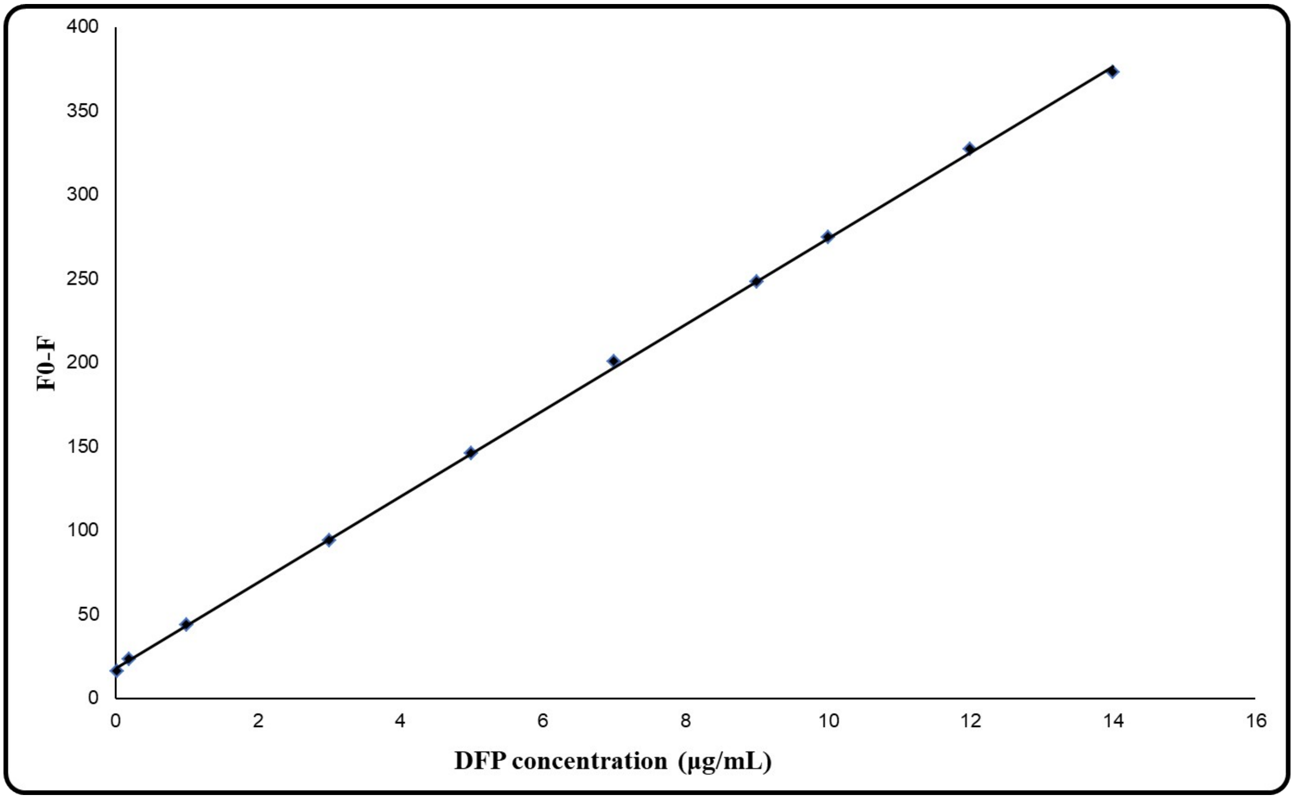

Fig. S8. Calibration curve for DFP.

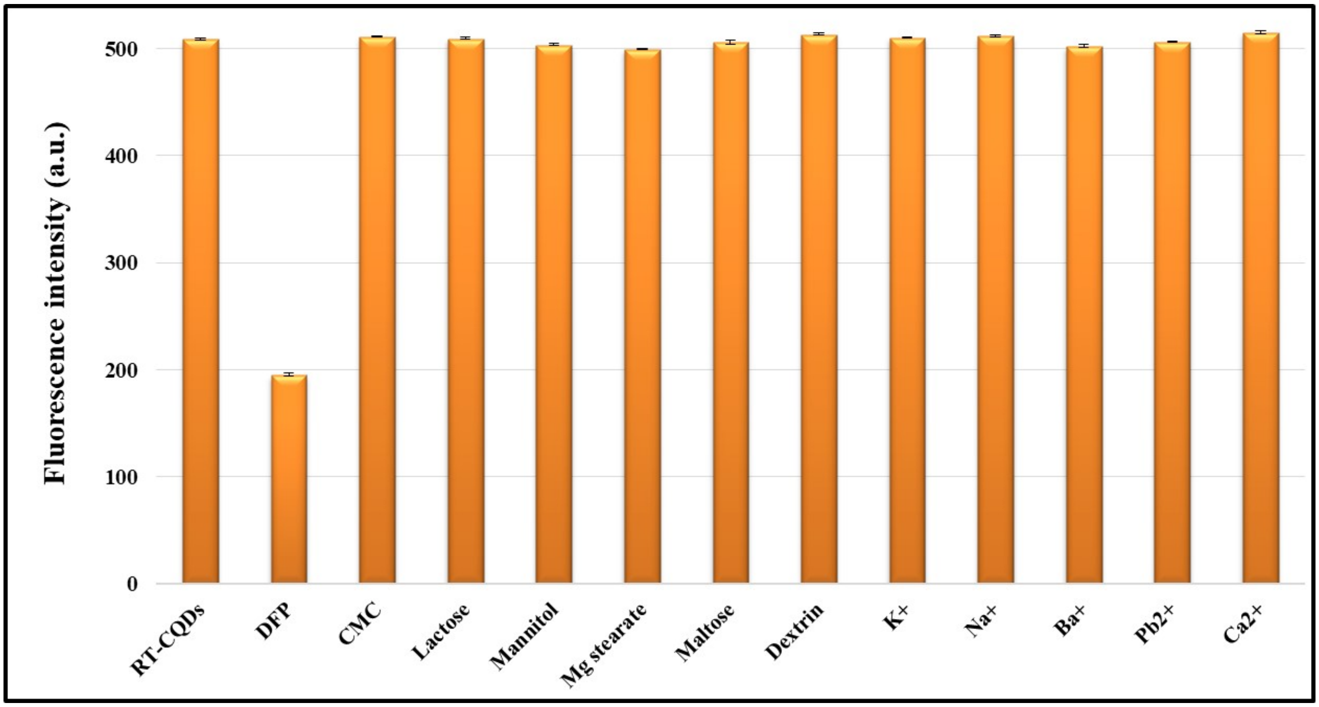

Fig. S9. Selectivity of RT-CQDs' nanosensor towards DFP (14.0 µg/mL) in the presence of interfering substances.

Table S1: Accuracy data of the proposed approach

Conc. taken (µg/mL)	Conc. found (µg/mL)	Mean Conc. found (µg/mL)	%Recovery*	Mean %Recovery ± SD
3.0	2.97<br>3.03<br>2.99	2.99	99.84	100.51 ± 0.62
7.0	7.13<br>7.05<br>7.05	7.07	101.06	
10.0	10.03<br>10.09<br>10.07	10.06	100.62	

* Each result is the average of three different measurements.

Table S2: Precision data of the proposed approach

Intra-day precision				Inter-day precision			
Conc. taken (µg/mL)	Mean Conc. Found* (µg/mL)	SD	%RSD	Conc. taken (µg/mL)	Mean Conc. found* (µg/mL)	SD	%RSD
3.0	2.98	1.28	1.29	3.0	2.99	0.62	0.61
7.0	8.08	0.17	0.17	7.0	7.09	0.90	0.89
10.0	10.12	0.13	0.13	10.0	10.13	1.23	1.21

* Each result is the average of three different concentrations.

Table S3: Robustness results of the proposed method

Variable	DFP	
**1. Buffer pH (7 ± 0.5)**	**%Recovery***	**%RSD**
6.5	99.09	0.48
7.0	100.31	0.13
7.5	99.16	0.93
**2. Volume of buffer (400.0 ± 5.0 µL)**	**% Recovery***	**%RSD**
395.0	101.11	0.73
400.0	100.31	0.13
405.0	101.76	0.63
**3. Volume of RT-CQDs (140.0 ± 5.0 µL)**	**% Recovery***	**%RSD**
135.0 µL	101.47	0.62
140.0 µL	100.49	0.13
145.0 µL	100.49	0.11

* Each result is the average of three replicates
